# Supplementary material for: The phosphatase DUSP22 inhibits UBR2-mediated K63-ubiquitination and activation of Lck downstream of TCR signalling
Source: Nat Commun. 2024 Jan 15;15:532. doi: 10.1038/s41467-024-44843-w (PMC10789758; doi:10.1038/s41467-024-44843-w)
Supplement: Supplementary file 1 — Supplementary Information [file 41467_2024_44843_MOESM1_ESM.pdf]

Supplemental Materials for

The phosphatase DUSP22 inhibits UBR2-mediated K63-ubiquitination and activation of Lck downstream of TCR signalling

The PDF file includes:

Supplementary Figures 1-8 and Supplementary Tables 1-2.

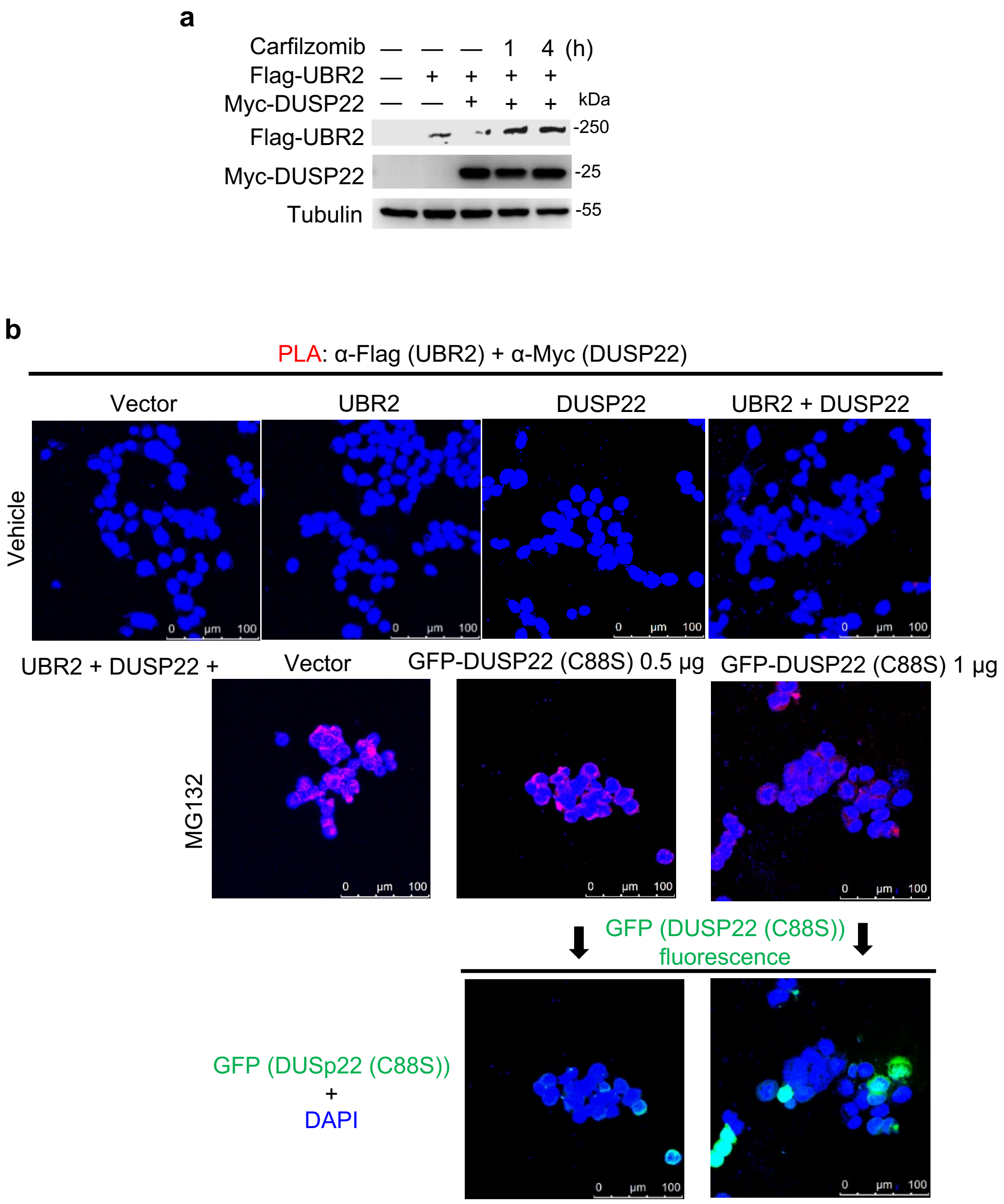

Supplementary Figure 1. DUSP22 (C88S) phosphatase-dead mutant proteins compete with DUSP22 proteins for UBR2 binding. a The proteasome inhibitor carfilzomib stabilized UBR2 protein levels in a time-dependent manner. Flag-UBR2 and Myc-DUSP22 plasmids were co-transfected into HEK293T cells. The cells were treated with 500 nM carfilzomib for the indicated time points and then subjected to immunoblotting analysis. **b** PLA data showed that DUSP22 (C88S) mutant overexpression decreased UBR2—DUSP22 interaction in HEK293T cells. Flag-UBR2, Myc-DUSP22, and either vector or GFP-DUSP22 (C88S) plasmids were co-transfected into HEK293T cells. Red fluorescence represents the interactions (< 40 nm) between Flag-UBR2 and Myc-DUSP22 proteins. GFP fluorescence (green color) indicates the expression of GFP-DUSP22 (C88S) in GFP-DUSP22 (C88S)-transfected cells. Images were captured with 400X original magnification by confocal microscope (Leica TCS SP5 II). Cell nuclei were stained with DAPI. Scale bar, 100 μm. The data shown are representatives of two independent experiments (a and b). Source data are provided as a Source Data file.

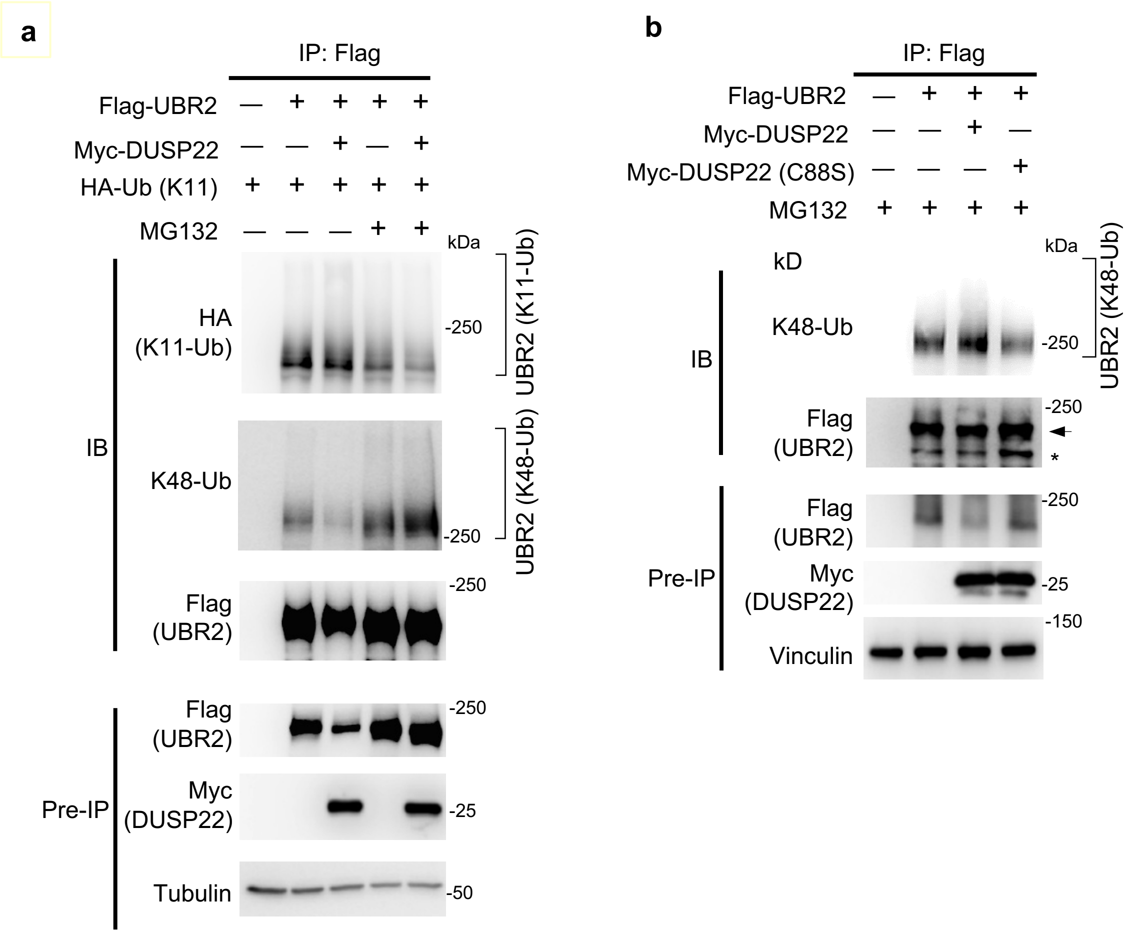

Supplementary Figure 2. DUSP22 induces UBR2 Lys48-linked ubiquitination but not Lys11-linked ubiquitination. a DUSP22 did not induce UBR2 Lys11-linked ubiquitination. HA-Lys11-only ubiquitin (K11-Ub) mutant, Flag-UBR2, and Myc-DUSP22 plasmids were co-transfected into HEK293T cells, followed by treatment with or without 25 μM MG132 for 4 h. The cell lysates were immunoprecipitated with anti-Flag antibody and then immunoblotted with anti-HA antibody, anti-ubiquitin (Lys48) antibody, or anti-Flag antibody. Immunoblotting of cell lysates before immunoprecipitation (Pre-IP) is shown at the bottom. **b** DUSP22 induced Lys48-linked ubiquitination, whereas the DUSP22 (C88S) phosphatase-dead mutant reduced UBR2 Lys48-linked ubiquitination. Flag-UBR2 plus either Myc-DUSP22 or Myc-DUSP22 (C88S) plasmids were co-transfected into HEK293T cells, followed by treatment with 25 μM MG132 for 4 h. The cell lysates were immunoprecipitated with anti-Flag antibody and then immunoblotted with anti-ubiquitin (Lys48) antibody or anti-Flag antibody. Immunoblotting of cell lysates before immunoprecipitation (Pre-IP) is shown at the bottom. The arrow denotes intact UBR2 protein, and asterisk denotes the UBR2 degraded protein. The data shown are representatives of three independent experiments (a and b). Source data are provided as a Source Data file.

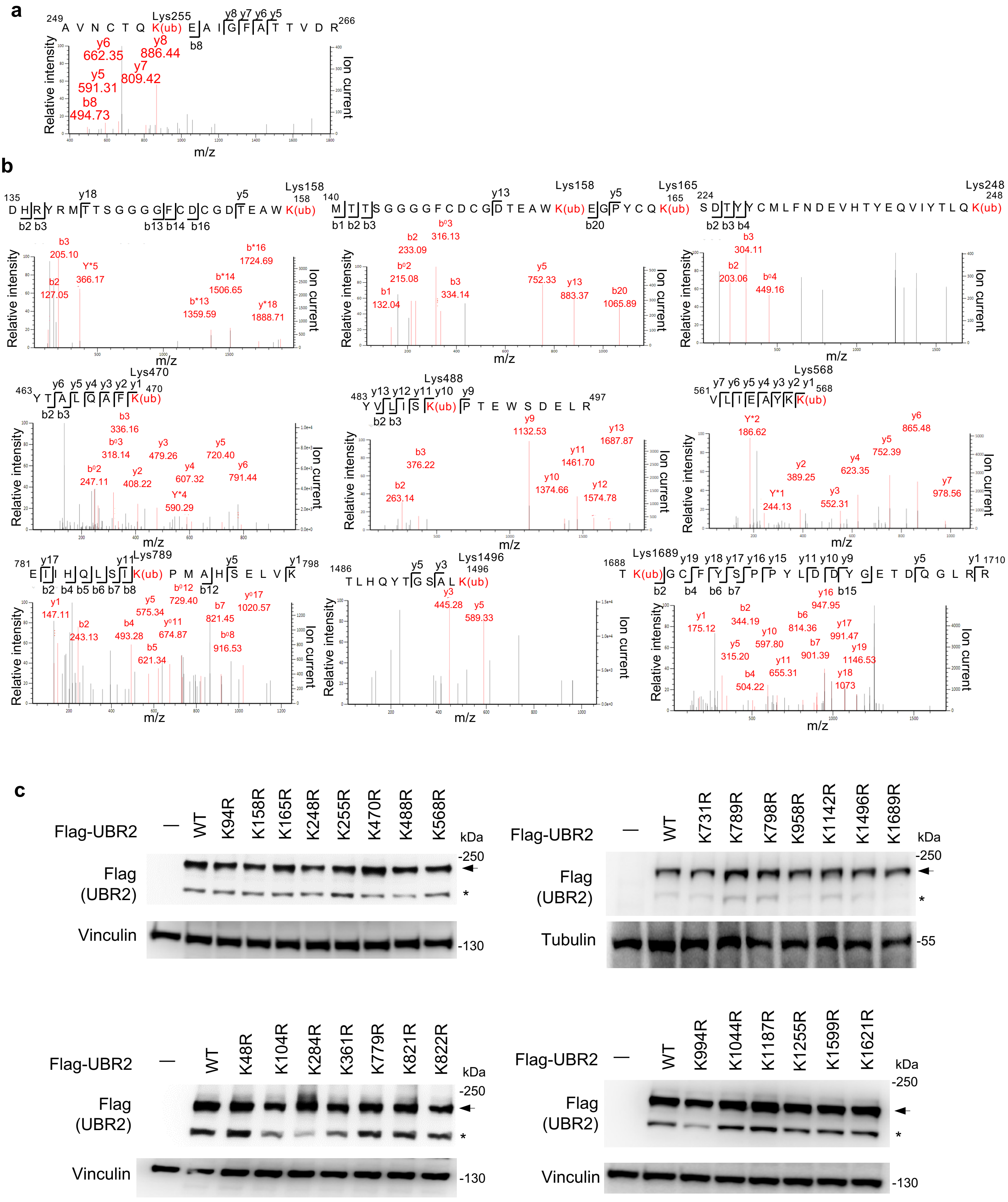
a
249 A V N C T Q K(ub) E A I G F A T T V D R 266
Lys255
Relative intensity
Ion current
m/z
b
Lys158
Lys165
Lys248
Lys470
Lys488
Lys568
Lys789
Lys1496
Lys1689
Relative intensity
Ion current
m/z
c
Flag-UBR2
— WT K94R K158R K165R K248R K255R K470R K488R K568R
Flag (UBR2)
Vinculin
kDa
-250
-130
— WT K731R K789R K798R K958R K1142R K1496R K1689R
Tubulin
-55
— WT K48R K104R K284R K361R K779R K821R K822R
— WT K994R K1044R K1187R K1255R K1599R K1621R
*

Supplementary Figure 3. Several ubiquitination sites of UBR2 are identified. a HEK293T cells were transfected with Flag-UBR2 plus Lys48-only ubiquitin mutant (K48-Ub) and with or without DUSP22 plasmids, followed by treatment with or without 25 μM MG132 for 4 h. Flag-tagged UBR2 proteins were immunoprecipitated with anti-Flag antibody and then subjected to mass spectrometry analysis. The ubiquitination sites of UBR2 and protein scores of UBR2 tryptic peptides containing the ubiquitinated residues are listed in (Supplementary Table 1). The MS/MS fragmentation spectra of UBR2 tryptic peptides containing the ubiquitinated residue Lys255 were showed. K(ub) denotes the ubiquitinated lysine residue. **b** The ubiquitination sites of UBR2 were identified by mass spectrometry analysis. Flag-UBR2 alone or Flag-UBR2 plus Myc-DUSP22 plasmids were co-transfected into HEK293T cells, followed by treatment (or no treatment) with 25 μM MG132 for 4 h. Flag-tagged UBR2 proteins were immunoprecipitated with anti-Flag antibody and then subjected to mass spectrometry analysis. The ubiquitination sites of UBR2 and protein scores of UBR2 tryptic peptides containing the ubiquitinated residues are listed in (Supplementary Table 2). The MS/MS fragmentation spectra of UBR2 tryptic peptides containing the ubiquitinated residue are showed. K(ub) denotes the ubiquitinated lysine residue. **c** The expression of individual UBR2 mutants alone in the absence of DUSP22 overexpression was comparable to that of wild-type (WT) UBR2. Individual Flag-UBR2 mutant plasmids were transfected into HEK293T cells. The cell lysates were subjected to immunoblotting using anti-Flag antibody. Immunoblotting of vinculin or tubulin was performed by reprobing the Flag (UBR2) immunoblot membrane with anti-vinculin or anti-tubulin antibody. The arrow denotes intact UBR2 protein, and asterisk denotes the UBR2 degraded protein. The data shown are representatives of three independent experiments (c). Source data are provided as a Source Data file.

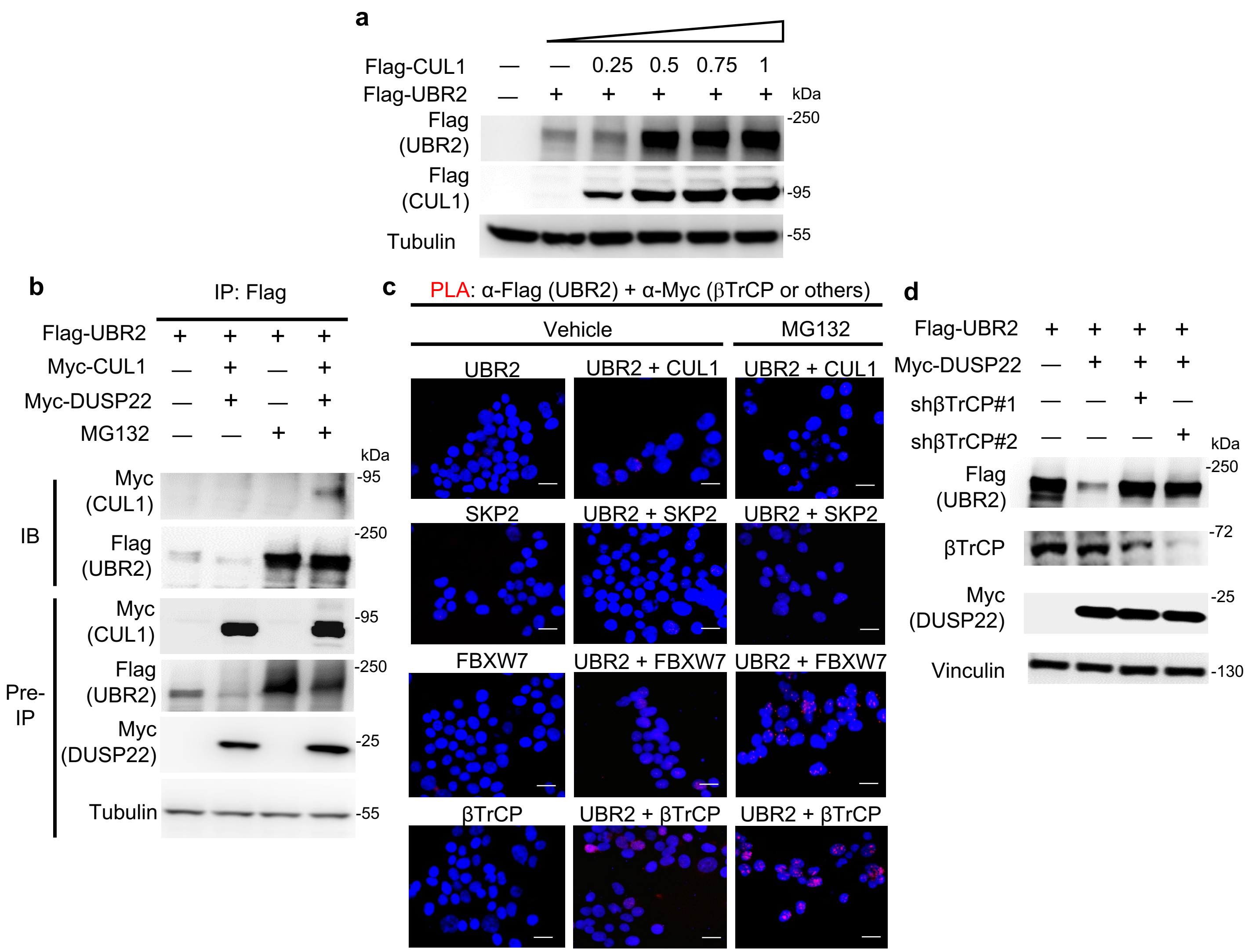

Supplementary Figure 4. CUL1 alone is unable to decrease UBR2 expression in the absence of DUSP22 overexpression. a Immunoblotting of Flag-tagged UBR2 (anti-Flag), Flag-tagged CUL1 (anti-Flag), and tubulin proteins from HEK293T cells co-transfected with Flag-UBR2 plus increasing amounts of Flag-CUL1 plasmids. **b** UBR2 interacted with CUL1. Flag-UBR2 plus either vector or Myc-CUL1 plasmids were co-transfected into HEK293T cells. Flag-tagged UBR2 proteins were immunoprecipitated with anti-Flag antibody and then immunoblotted with anti-Myc or anti-Flag antibody. Anti-tubulin immunoblotting was performed by reprobing the anti-Myc (DUSP22) immunoblot membrane with anti-tubulin antibody. **c** Proximity ligation assay (PLA) showed the UBR2—βTrCP interaction in HEK293T cells. Flag-UBR2 plus individual Myc-CUL1, Myc-SKP2, Myc-FBXW7, and Myc-βTrCP plasmids were co-transfected into HEK293T cells. Red fluorescence represents interactions (< 40 nm) between indicated protein pairs. Images were captured with 400X original magnification by Leica DM2500 fluorescence microscope. Cell nuclei were stained with DAPI. Scale bar, 10 μm. **d** βTrCP shRNA knockdown blocked DUSP22-induced UBR2 degradation. Flag-UBR2, Myc-DUSP22, and individual βTrCP shRNAs were co-transfected into HEK293T cells. The levels of Flag-tagged UBR2 proteins were determined by immunoblotting analysis. Anti-vinculin immunoblotting was performed by reprobing the anti-Flag (UBR2) immunoblot membrane with anti-vinculin antibody. The data shown are representatives of three independent experiments (a-d). Source data are provided as a Source Data file.

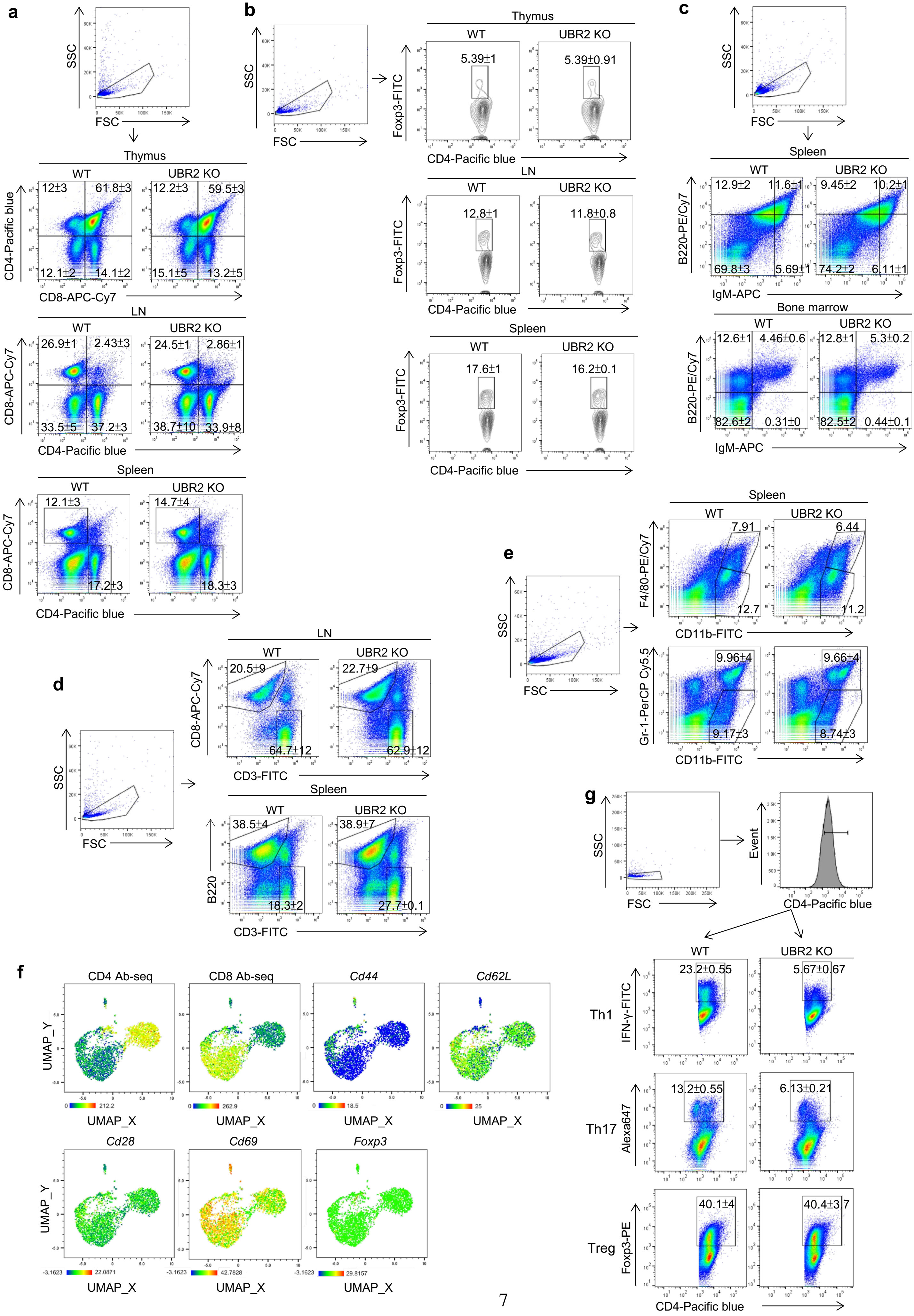
a
SSC
FSC
Thymus
WT
UBR2 KO
12±3
61.8±3
12.1±2
14.1±2
12.2±3
59.5±3
15.1±5
13.2±5
CD4-Pacific blue
CD8-APC-Cy7
LN
26.9±1
2.43±3
33.5±5
37.2±3
24.5±1
2.86±1
38.7±10
33.9±8
CD8-APC-Cy7
CD4-Pacific blue
Spleen
12.1±3
17.2±3
14.7±4
18.3±3
b
Thymus
5.39±1
5.39±0.91
Foxp3-FITC
CD4-Pacific blue
LN
12.8±1
11.8±0.8
Spleen
17.6±1
16.2±0.1
c
Spleen
12.9±2
11.6±1
69.8±3
5.69±1
9.45±2
10.2±1
74.2±2
6.11±1
B220-PE/Cy7
IgM-APC
Bone marrow
12.6±1
4.46±0.6
82.6±2
0.31±0
12.8±1
5.3±0.2
82.5±2
0.44±0.1
d
LN
20.5±9
64.7±12
22.7±9
62.9±12
CD8-APC-Cy7
CD3-FITC
Spleen
38.5±4
18.3±2
38.9±7
27.7±0.1
B220
e
7.91
12.7
6.44
11.2
F4/80-PE/Cy7
CD11b-FITC
9.96±4
9.17±3
9.66±4
8.74±3
Gr-1-PerCP Cy5.5
f
CD4 Ab-seq
CD8 Ab-seq
Cd44
Cd62L
Cd28
Cd69
Foxp3
UMAP_Y
UMAP_X
g
Event
Th1
23.2±0.55
5.67±0.67
IFN-γ-FITC
Th17
13.2±0.55
6.13±0.21
Alexa647
Treg
40.1±4
40.4±3.7
Foxp3-PE

Supplementary Figure 5. Normal T cell development in thymus of UBR-deficient mice. a and b Flow cytometry analyses of T cells (a) and Treg ($CD4^+$ $Foxp3^+$-double positive) cells (b) from the thymus, spleen, or lymph nodes of 5-week-old wild-type (WT) or UBR2 knockout mice. **c** Flow cytometry analyses of $B220^+$ B cells from the spleen or bone marrow of WT or UBR2 knockout mice. **d** Flow cytometry analyses of $B220^+$ or $CD3^+$ cells from the spleen or lymph nodes of wild-type (WT) or UBR2 knockout mice. **e** Flow cytometry analyses of $F4/80^+CD11b^+$ double-positive cells or Gr-1^+CD11b^+ double- positive cells from spleen of wild-type (WT) or UBR2 knockout mice. **f** UMAP plots display the expression levels of individual cell makers in murine T cells of wild-type and UBR2 knockout mice. The transcripts were subjected to UMAP dimension reduction and clustering analysis by BD Rhapsody software. The gene expression levels of individual cell markers in each T cell were shown in the indicated color scheme. Ab-seq denotes Ab-Oligos (antibody-oligonucleotides). **g** *In vitro* Th1, Th17, and Treg differentiation of $CD4^+$ splenic T cells from wild-type (WT) and UBR2 knockout mice. IFN-γ-, IL-17A-, or Foxp3-positive $CD4^+$ T cells were analyzed by flow cytometry. The data shown are representatives of two independent experiments (a, b, c, d, e and g).

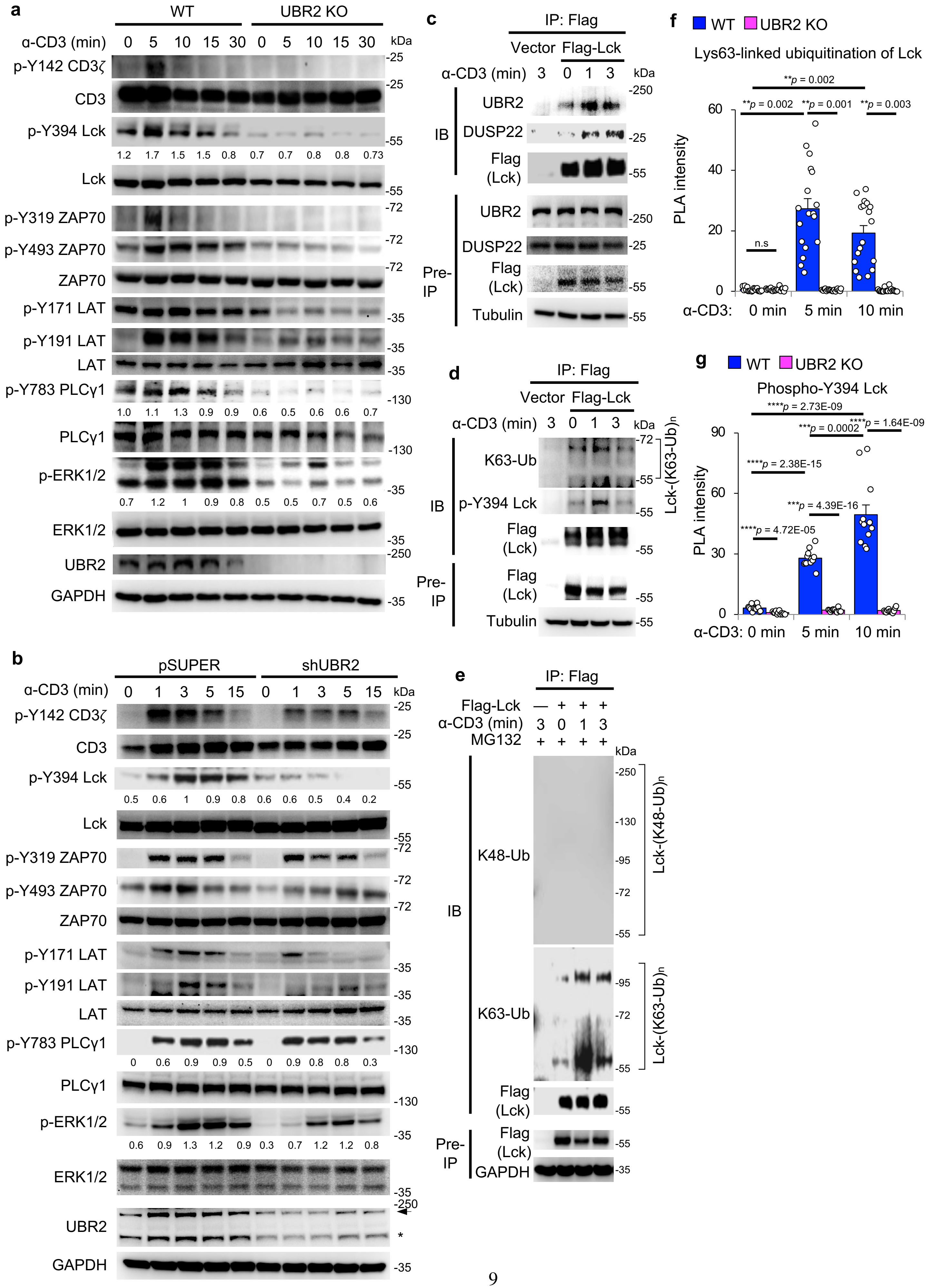
a
WT
UBR2 KO
α-CD3 (min) 0 5 10 15 30 0 5 10 15 30 kDa
p-Y142 CD3ζ
CD3
p-Y394 Lck
1.2 1.7 1.5 1.5 0.8 0.7 0.7 0.8 0.8 0.73
Lck
p-Y319 ZAP70
p-Y493 ZAP70
ZAP70
p-Y171 LAT
p-Y191 LAT
LAT
p-Y783 PLCγ1
1.0 1.1 1.3 0.9 0.9 0.6 0.5 0.6 0.6 0.7
PLCγ1
p-ERK1/2
0.7 1.2 1 0.9 0.8 0.5 0.5 0.7 0.5 0.6
ERK1/2
UBR2
GAPDH
b
pSUPER
shUBR2
α-CD3 (min) 0 1 3 5 15 0 1 3 5 15 kDa
p-Y142 CD3ζ
CD3
p-Y394 Lck
0.5 0.6 1 0.9 0.8 0.6 0.6 0.5 0.4 0.2
Lck
p-Y319 ZAP70
p-Y493 ZAP70
ZAP70
p-Y171 LAT
p-Y191 LAT
LAT
p-Y783 PLCγ1
0 0.6 0.9 0.9 0.5 0 0.9 0.8 0.8 0.3
PLCγ1
p-ERK1/2
0.6 0.9 1.3 1.2 0.9 0.3 0.7 1.2 1.2 0.8
ERK1/2
UBR2
*
GAPDH
c
IP: Flag
Vector Flag-Lck
α-CD3 (min) 3 0 1 3 kDa
IB
UBR2
DUSP22
Flag (Lck)
Pre-IP
UBR2
DUSP22
Flag (Lck)
Tubulin
d
IP: Flag
Vector Flag-Lck
α-CD3 (min) 3 0 1 3 kDa
IB
K63-Ub
p-Y394 Lck
Flag (Lck)
Lck-(K63-Ub)n
Pre-IP
Flag (Lck)
Tubulin
e
IP: Flag
Flag-Lck — + + +
α-CD3 (min) 3 0 1 3
MG132 + + + +
kDa
IB
K48-Ub
Lck-(K48-Ub)n
K63-Ub
Lck-(K63-Ub)n
Flag (Lck)
Pre-IP
Flag (Lck)
GAPDH
f
WT UBR2 KO
Lys63-linked ubiquitination of Lck
**p = 0.002
**p = 0.002
**p = 0.001
**p = 0.003
n.s
PLA intensity
α-CD3: 0 min 5 min 10 min
g
WT UBR2 KO
Phospho-Y394 Lck
****p = 2.73E-09
***p = 0.0002
****p = 1.64E-09
****p = 2.38E-15
***p = 4.39E-16
****p = 4.72E-05
PLA intensity
α-CD3: 0 min 5 min 10 min

h

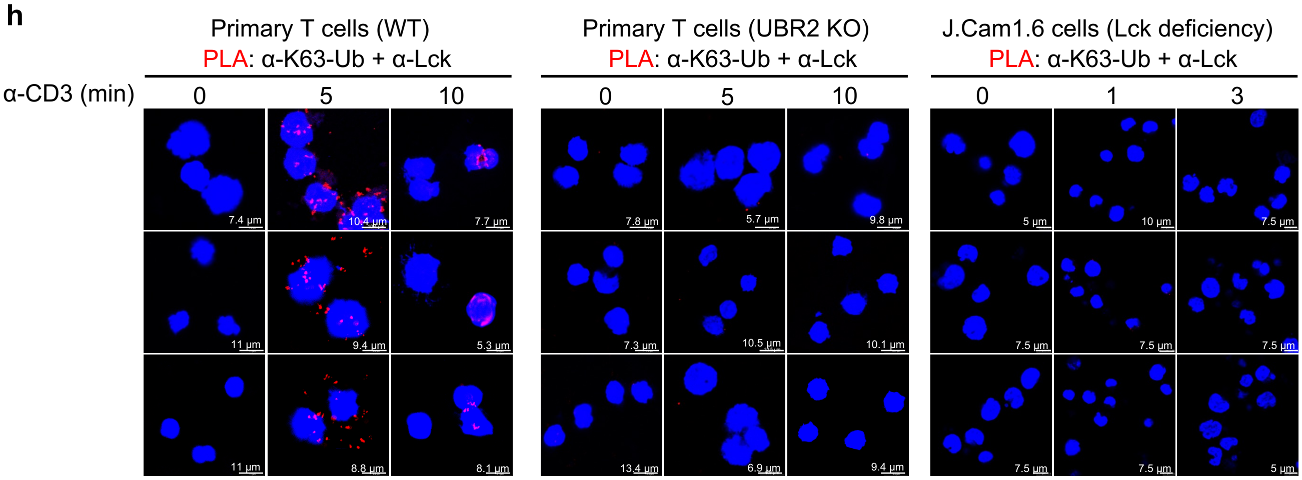

i

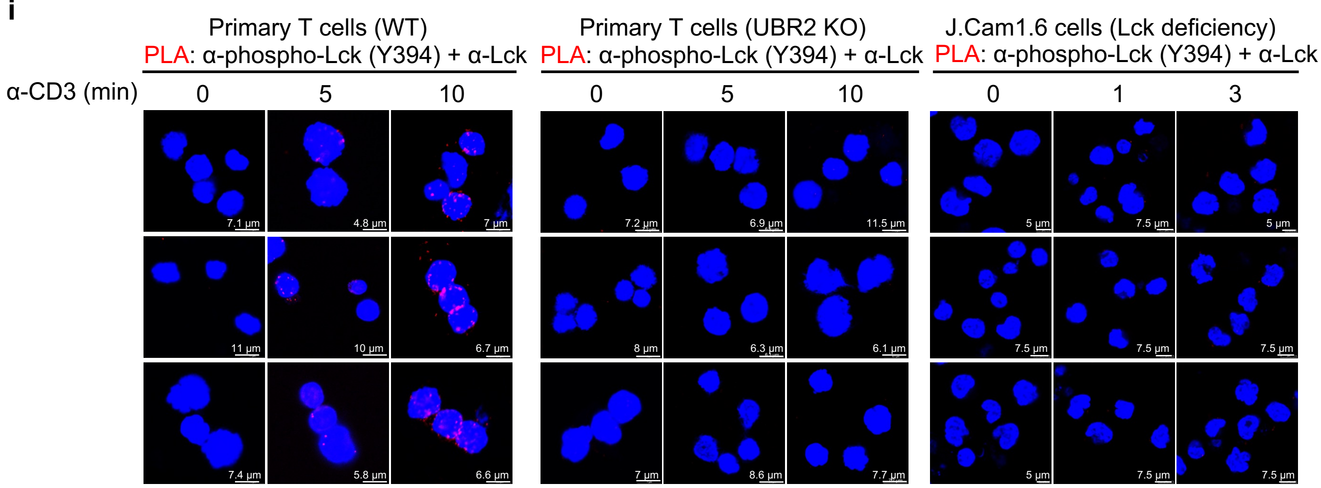

Supplementary Figure 6. UBR2 interacts with Lck or Lck (K99/276R) mutant. a and **b** TCR signaling was deaccelerated by UBR2 knockout and UBR2 knockdown. The levels of various TCR signaling molecules in purified wild-type and UBR2-knockout murine primary T cells from lymph node and spleen or shRNA transfected Jurkat T cells on anti-CD3 stimulation were measured by immunoblotting. The phosphorylation levels of TCR signaling molecules were detected using anti-phospho-CD3ζ (Tyr142), anti-phospho-Lck (Tyr394), anti-phospho-ZAP70 (Tyr319), anti-phospho-ZAP70 (Tyr493), anti-phospho-LAT (Tyr171), anti-phospho-LAT (Tyr191), anti-phospho-PLCγ1 (Tyr783), and anti-phospho-ERK1/2 antibodies. Quantification of p-Lck, p-PLCγ, and p-ERK proteins (normalized to their total-form proteins) is shown at the bottom of individual panels. **c** Lck interacted with UBR2 and DUSP22. Immunoprecipitation and immunoblotting analyses of UBR2, DUSP22, and Lck proteins in Jurkat T cells transfected with Flag-Lck plasmid. T cells were stimulated with anti-CD3 antibody at indicated time periods. **d** TCR signaling stimulated Lys63-linked ubiquitination and Tyr394 phosphorylation of Lck. Flag-Lck plasmid was transfected into Jurkat T cells, followed by anti-CD3 stimulation. Flag-tagged Lck proteins were immunoprecipitated with anti-Flag antibody and then immunoblotted with anti-ubiquitin (Lys63) antibody, anti-phospho-Lck (Tyr394) antibody, anti-Flag antibody, or anti-tubulin antibody. **e** TCR signaling stimulated Lys63-linked, but not Lys48-linked, ubiquitination of Lck. Flag-Lck plasmid was transfected into Jurkat T cells, followed by 20 μM MG132 for 1 h. T cells were then stimulated with anti-CD3 antibody for indicated time period. Flag-tagged Lck proteins were immunoprecipitated with anti-Flag antibody and then immunoblotted with anti-ubiquitin (Lys48) antibody, anti-ubiquitin (Lys63) antibody, anti-Flag antibody, or anti-GAPDH antibody.

f Quantification of Lys63-linked ubiquitination of Lck fluorescence intensity. The red signal intensity of fig. 6c (upper panel) were plotted. Experiments were run 4 independent times with an n = 18. Data are presented as mean values ± SEM. Significance was determined by a 2-tailed student's t test with *, $p < 0.05$; **, $p < 0.01$; ***, $p < 0.001$, ****, $p < 0.0001$. n.s., not significant. **g** Quantification of phosphorylated Tyr394 of Lck fluorescence intensity. The red signal intensity of Fig. 6c (lower panel) was plotted. Experiments were run 4 independent times with an n = 12. Data are presented as mean values ± SEM. Significance was determined by a 2-tailed student's t test with *, $p < 0.05$; **, $p < 0.01$; ***, $p < 0.001$, ****, $p < 0.0001$. **h and i** PLA assays of ubiquitinated and phosphorylated Lck in TCR-stimulated T cells of wild-type (WT) or UBR2 knockout mice and Lck-deficient Jurkat (J.Cam1.6 clone) T cells using anti-Lck antibody plus either anti-ubiquitin (Lys63) or anti-phospho-Lck (Tyr394) antibodies. Red fluorescence represents the endogenous Lck proteins containing Lys63-linked ubiquitination or Tyr394 phosphorylation. Images of different fields were captured by confocal microscope (Leica TCS SP5 II). Cell nuclei were stained with DAPI. The data shown are representatives of two independent experiments (a and b). The data shown are representatives of at least three independent experiments (c, d, e. h, and i). Source data are provided as a Source Data file.

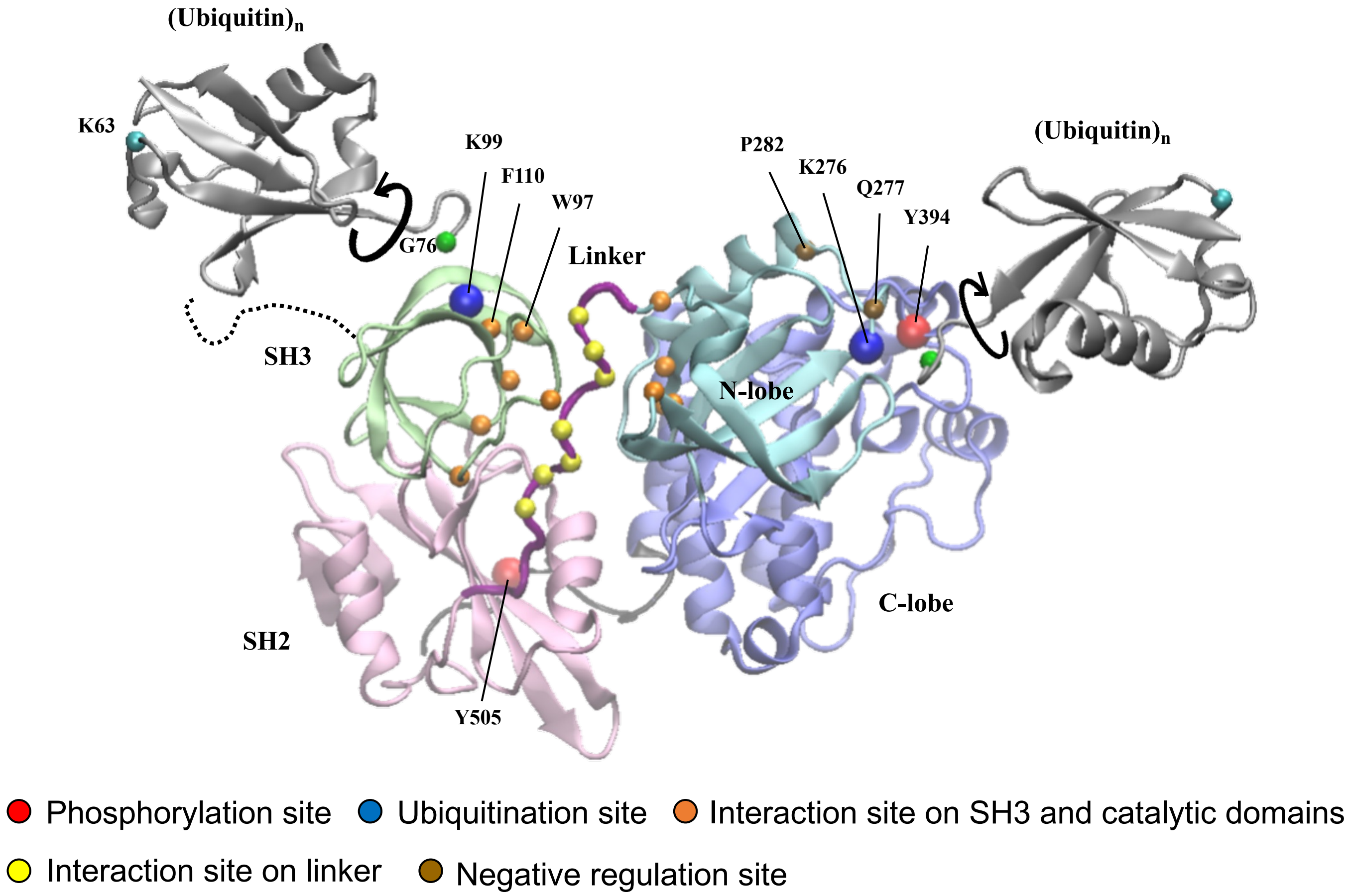

Supplementary Figure 7. The modeled Lck protein with ubiquitins conjugating to Lys99 and Lys276 residues. The Lck protein contains a linker (purple coil, 224 to 237 a.a.) and three domains, including SH3 domain (light green ribbon, 63 to 121 a.a.), SH2 domain (pink ribbon, 122 to 223 a.a.), and the catalytic domain. The catalytic domain contains two lobes, N-lobe (light blue ribbon, 238 to 319 a.a.) and C-lobe (blue ribbon, 320 to 498 a.a.). The linker connects the catalytic domain with the SH2 domain and SH3 domain. The Cα atoms from amino acids on the SH3 domain and catalytic domain that interact with the linker are marked by orange spheres. The interaction sites on the linker are marked by yellow spheres. The Cα atoms of the ubiquitination sites, Lys99 and Lys276 residues are labeled by blue spheres. The Gly76 residue (green sphere) of ubiquitin (gray ribbon) is conjugated to the Lck Lys99 residue. The ubiquitination site Lys99 residue is near the interaction sites (Trp97 and Phe110 residues) on the SH3 domain. Another ubiquitination site Lys276 is close to Gln277, Pro282, and Tyr394 residues. The phosphorylation sites, Tyr394 and Tyr505 residues, are labeled by red spheres.

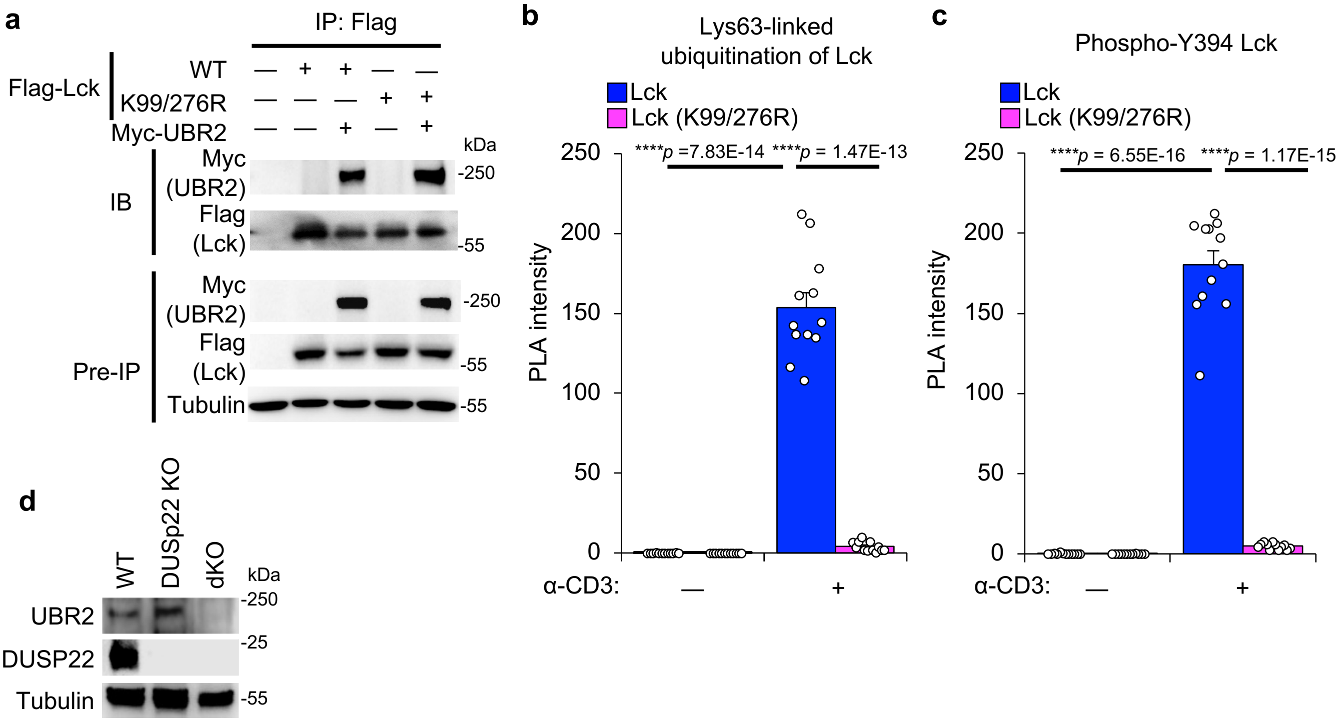

Supplementary Figure 8. Lys63-linked ubiquitination at Lys99 and Lys276 residues is required for Lck Tyr394 phosphorylation. a Coimmunoprecipitation and immunoblotting analyses of UBR2, Lck, and Lck (K99/276R) mutant proteins in HEK293T cells co-transfected with Myc-UBR2 plasmid plus either Flag-Lck or Flag-Lck (K99/276R) mutant plasmids. **b** Lys63-linked ubiquitination of Lck fluorescence intensity. The red signal intensity of Fig. 6h (upper panel) were plotted. **c** Quantification of phosphorylated Tyr394 of Lck fluorescence intensity. The red signal intensity of Fig. 6h (lower panel) was plotted. The analyses are means ± SEM of three independent experiments (b and c). **d.** Immunoblotting analyses of UBR2 and DUSP22 protein levels in peripheral blood T cells of wild-type (WT), DUSP22 knockout (KO), and DUSP22/UBR2 double knockout (dKO) mice. The data shown are representatives of three independent experiments (a and d). Experiments were run 3 independent times with an n = 12 (b and c). Data are presented as mean values ± SEM. Significance was determined by a 2-tailed student's t test with *, $p < 0.05$; **, $p < 0.01$; ***, $p < 0.001$, ****, $p < 0.0001$. Source data are provided as a Source Data file.

UBR2 + K48-Ub		UBR2 + DUSP22 + K48-Ub		UBR2 + DUSP22 + K48-Ub + MG132	
Ubiquitination site	Protein score	Ubiquitination site	Protein score	Ubiquitination site	Protein score
Lys94	ND	Lys94	ND	Lys94	11*
Lys255	9*	Lys255	ND	Lys255	ND

Supplementary Table 1. The ubiquitination sites of UBR2. Mass spectrometry analysis identified UBR2 ubiquitination sites from HEK293T cells transfected with Flag-UBR2 plus Lys48-only ubiquitin mutant (K48-Ub) and with or without DUSP22 plasmids, followed by treatment (or no treatment) with 25 μM MG132 for 4 h. Flag-tagged UBR2 proteins were immunoprecipitated with anti-Flag antibody and then subjected to mass spectrometry analysis. The ubiquitination sites of UBR2 and protein scores of UBR2 tryptic peptides containing the ubiquitinated residue are listed. Asterisk denotes the highest protein score of the peptide in mass spectrometry analysis. The MS/MS fragmentation spectra of UBR2 tryptic peptides containing the ubiquitinated residue Lys255 were showed in Supplementary Fig. 3b.

UBR2		UBR2 + DUSP22 + MG132		UBR2 + MG132	
Ubiquitination site	Protein score	Ubiquitination site	Protein score	Ubiquitination site	Protein score
Lys158	7	Lys158	11*	Lys158	ND
Lys165	ND	Lys165	64*	Lys165	49
Lys248	ND	Lys248	ND	Lys248	2
Lys470	ND	Lys470	15	Lys470	19*
Lys488	ND	Lys488	33*	Lys488	19
Lys568	ND	Lys568	47*	Lys568	34
Lys789	ND	Lys789	ND	Lys789	6*
Lys1496	ND	Lys1496	ND	Lys1496	9*
Lys1689	90	Lys1689	80	Lys1689	92*

Supplementary Table 2. The ubiquitination sites of UBR2. Mass spectrometry analysis identified UBR2 ubiquitination sites from HEK293T cells transfected with Flag-UBR2 alone or Flag-UBR2 plus Myc-DUSP22 plasmids, followed by treatment (or no treatment) with 25 μM MG132 for 4 h. Flag-tagged UBR2 proteins were immunoprecipitated with anti-Flag antibody and then subjected to mass spectrometry analysis. The ubiquitination sites of UBR2 and protein scores of UBR2 tryptic peptides containing the ubiquitinated residues are listed. Asterisk denotes the highest protein score of the peptide in the mass spectrometry analysis. The MS/MS fragmentation spectra of UBR2 tryptic peptides containing the ubiquitinated residue are showed in Supplementary Fig. 3b.
