# Supplementary material for: The phosphatase DUSP22 inhibits UBR2-mediated K63-ubiquitination and activation of Lck downstream of TCR signalling
Source: Nat Commun. 2024 Jan 15;15:532. doi: 10.1038/s41467-024-44843-w (PMC10789758; doi:10.1038/s41467-024-44843-w)
Supplement: Supplementary file 3 — Reporting Summary [file 41467_2024_44843_MOESM3_ESM.pdf]

nature portfolio

Corresponding author(s): Huai-Chia Chuang and Tse-Hua Tan

Last updated by author(s): Dec 16, 2023

Reporting Summary

Nature Portfolio wishes to improve the reproducibility of the work that we publish. This form provides structure for consistency and transparency in reporting. For further information on Nature Portfolio policies, see our Editorial Policies and the Editorial Policy Checklist.

Statistics

For all statistical analyses, confirm that the following items are present in the figure legend, table legend, main text, or Methods section.

n/a	Confirmed	
☐	☒	The exact sample size (*n*) for each experimental group/condition, given as a discrete number and unit of measurement
☐	☒	A statement on whether measurements were taken from distinct samples or whether the same sample was measured repeatedly
☐	☒	The statistical test(s) used AND whether they are one- or two-sided<br>*Only common tests should be described solely by name; describe more complex techniques in the Methods section.*
☒	☐	A description of all covariates tested
☒	☐	A description of any assumptions or corrections, such as tests of normality and adjustment for multiple comparisons
☐	☒	A full description of the statistical parameters including central tendency (e.g. means) or other basic estimates (e.g. regression coefficient) AND variation (e.g. standard deviation) or associated estimates of uncertainty (e.g. confidence intervals)
☐	☒	For null hypothesis testing, the test statistic (e.g. *F*, *t*, *r*) with confidence intervals, effect sizes, degrees of freedom and *P* value noted<br>*Give P values as exact values whenever suitable.*
☒	☐	For Bayesian analysis, information on the choice of priors and Markov chain Monte Carlo settings
☒	☐	For hierarchical and complex designs, identification of the appropriate level for tests and full reporting of outcomes
☒	☐	Estimates of effect sizes (e.g. Cohen's *d*, Pearson's *r*), indicating how they were calculated

Our web collection on statistics for biologists contains articles on many of the points above.

Software and code

Policy information about availability of computer code

Data collection	For proteomics, Gel-extracted proteins were digested with trypsin and subjected to LC–MS/MS analyses by LTQ-Orbitrap Elite hybrid mass spectrometer (for identifying DUSP22-interacting proteins and UBR2-interacting protein) or LTQ-Orbitrap Fusion hybrid mass spectrometer (for mapping UBR2 phosphorylated residues, UBR2 ubiquitinated residues, and Lck ubiquitinated residues) using approaches described previously (Cancer Res. 79, 4978-4993 (2019); EMBO Mol. Med. 14, e15904 (2022)). The peptide data were analyzed by MASCOT MS/MS Ions Search (Matrix Science). FFor fluorescent signal in cells, images were detected by fluorescence microscope (DM2500; Leica Microsystems, Buffalo Grove, IL, USA) or confocal microscope Leica TCS SP5, and Leica TCS SP5 II. Images were analyzed by LAS X software (v3.7.4). For scRNA-seq, single-cell capture and cDNA synthesis were performed using the BD Rhapsody Single-Cell Analysis System (210966 v1.0). cDNA libraries were constructed combined with Sample Tag and BD AbSeq libraries (BD Biosciences, 23-21752-00). cDNA libraries of individual cells were constructed using BD Rhapsody WTA Amplification Kit (Cat. 633801). Paired-end sequencing was performed on a HiSeq X Ten sequencer (Illumina). Data analysis and quality control were performed following the BD Biosciences Rhapsody pipeline. Uniform Manifold Approximation and Projection (UMAP) generation were conducted with the R package Seurat (v3.0). For flow cytometry, data were acquired with a FACS CantoII (BD Biosciences) and analyzed with FlowJo (v10.8.1) analytical software. For immunoblotting, signals were detected by Syngene and analyzed by Pxi9 Access GENESys software. Statistical analyses were performed by using Excel (v16.7) or BD SEQGEQ (v1.8.0).
Data analysis	Mass spectrometry data was analyzed using MASCOT MS/MS Ions Search (Matrix Science). The PLA signals and immunoblotting were quantified by image J (v1.53t). For scRNA-seq analysis, marker identification and UMAP generation were used by the R package Seurat (v3.0). Immunoblotting images were performed by Pxi9 Access GENESys software. Flow cytometry data were analyzed with FlowJo software (v10.8.1). Statistical analyses were performed by using Excel (v16.7) or BD SEQGEQ (v1.8.0).

For manuscripts utilizing custom algorithms or software that are central to the research but not yet described in published literature, software must be made available to editors and reviewers. We strongly encourage code deposition in a community repository (e.g. GitHub). See the Nature Portfolio guidelines for submitting code & software for further information.

Data

Policy information about availability of data

All manuscripts must include a data availability statement. This statement should provide the following information, where applicable:

- Accession codes, unique identifiers, or web links for publicly available datasets
- A description of any restrictions on data availability
- For clinical datasets or third party data, please ensure that the statement adheres to our policy

All data are available in the main text or the supplementary materials. The raw single cell RNA sequencing data generated in this study have been deposited in the Sequence Read Archive database under accession code of SRR25120110 [https://www.ncbi.nlm.nih.gov/sra/?term=SRR25120110]. The raw proteomic mass data generated in this study have been deposited in the ProteomeXchange Consortium via the PRIDE proteomics IDEntifications database under accession codes of PXD043490 [https://www.ebi.ac.uk/pride/archive/projects/PXD043490/private], PXD043457 [https://www.ebi.ac.uk/pride/archive/projects/PXD043457/private], PXD043454 [https://www.ebi.ac.uk/pride/archive/projects/PXD043454/private] and PXD043490 [https://www.ebi.ac.uk/pride/archive/projects/PXD043490/private]. The protein structure was modeled by modeled by SWISS-MODEL web server and visulized by Visual Molecular Dynamics (VMD) molecular visualization program (http://www.ks.uiuc.edu/). The chicken Src structure (PDB ID 2PTK) was dowload from [https://www.rcsb.org/structure/2PTK]. The ubiquitin structure (PDB ID 3HMH) was downloaded from RSCB PDB [https://www.rcsb.org/structure/3HMH].

Research involving human participants, their data, or biological material

Policy information about studies with human participants or human data. See also policy information about sex, gender (identity/presentation), and sexual orientation and race, ethnicity and racism.

Reporting on sex and gender	The term gender is not used. Patient sex was not considered in the study design.
Reporting on race, ethnicity, or other socially relevant groupings	n/a
Population characteristics	A total of 12 individuals, including 6 healthy individuals (age from 24 to 49 year old) and 6 SLE patients (age from 24 to 49 year old) were enrolled in this study.
Recruitment	For collecting peripheral blood samples, 6 health individuals and 6 SLE patients who underwent clinic were invited to participate in academic research by providing peripheral blood samples. The selection of SLE patients was based on a well-defined SLE diagnosis.
Ethics oversight	This study was conducted in accordance with the Helsinki Declaration. For collecting peripheral blood samples, 6 health individuals and 6 SLE patients who underwent clinic were invited to participate in academic research by providing peripheral blood samples. The selection of SLE patients was based on a well-defined SLE diagnosis. The peripheral blood collection, T-cell purification, and the experiments were approved by the ethics committees of Taipei Veterans General Hospital (2017-06-003BC) and National Taiwan University Hospital (109-008-E). All study participants provided written informed consent prior to enrollment.

Note that full information on the approval of the study protocol must also be provided in the manuscript.

Field-specific reporting

Please select the one below that is the best fit for your research. If you are not sure, read the appropriate sections before making your selection.

☒ Life sciences ☐ Behavioural & social sciences ☐ Ecological, evolutionary & environmental sciences

For a reference copy of the document with all sections, see nature.com/documents/nr-reporting-summary-flat.pdf

Life sciences study design

All studies must disclose on these points even when the disclosure is negative.

Sample size	Sample sizes were determined from similar experiments in the literatures of Li et al Nature Communications, 5, 1-13 (2014), Chuang et al Nature Communications 5, 4602 (2014), and Chuang et al 12, 1113–1118 (2011). Sample sizes and the number of independent experiments are stated in the Statistical analysis and reproducibility section of Methods. Three or more independent results were used to for statistical analysis.
Data exclusions	No data were excluded.
Replication	The experimental results were consistently reproduced, and the figure legends specify the exact number (n) of biological replicates used in the study.
Randomization	All samples of the same genotype were randomly assigned for the control and experimental groups, and all cells analyzed were randomly selected from in vivo and in vitro samples.

Blinding

For mass spectrometry analysis, animal studies, scRNA sequencing, and cytokine measurements were carrierd out by an operator who was blinded to experiment groups.

Reporting for specific materials, systems and methods

We require information from authors about some types of materials, experimental systems and methods used in many studies. Here, indicate whether each material, system or method listed is relevant to your study. If you are not sure if a list item applies to your research, read the appropriate section before selecting a response.

Materials & experimental systems

n/a	Involved in the study
☐	☒ Antibodies
☐	☒ Eukaryotic cell lines
☒	☐ Palaeontology and archaeology
☐	☒ Animals and other organisms
☐	☒ Clinical data
☒	☐ Dual use research of concern
☒	☐ Plants

Methods

n/a	Involved in the study
☒	☐ ChIP-seq
☐	☒ Flow cytometry
☒	☐ MRI-based neuroimaging

Antibodies

Antibodies used

Anti-Flag (clone M2, Cat F3165, Lot SLCJ3741), anti-Myc (clone 9E10, Cat M4439), anti-Lys48-specific ubiquitin (clone Apu2, Cat 05-1307, Lot ZRB2150), anti-Lys63-specific ubiquitin (clone HWA4C4, Cat 05-1313, Lot 3147606), anti-vinculin (clone VIIF9, Cat MAB3574, Lot 3951156), anti-GST (clone DG122-2A7, Cat 05-311), and anti-His (clone 6AT18, Cat SAB1305538, Lot SA141112AB) monoclonal antibodies were purchased from Sigma-Aldrich. Anti-CUL1 (clone D5, Cat sc-17775, Lot B0714), and anti-CD3 zeta (clone 6B10.2, Cat sc-1239, Lot J1507) antibodies were purchased from Santa Cruze Biotechnology. Anti-UBR2 (Cat PA5-37161, Lot VG3023905) and anti-tubulin (clone BT7R, Cat MA5-16308, Lot QG218956) antibodies were purchased from Thermo Fisher Scientific. Anti-HA (clone C29F4, Cat 3724S, Lot 8), anti-phospho-ZAP70 (Y319) (Cat 2701, Lot 7), anti-phospho-ZAP70 (Y493) (Cat 2704, Lot 3), anti-ZAP70 (clone 99F2, Cat 2705, Lot 2), anti-phospho-LAT (Y171) (Cat 3581, Lot 1), anti-phospho-PLCγ1 (Y783) (Cat 2821, Lot 7), anti-βTrCP (clone D13F10, Cat 4394, Lot 4) and anti-Lck (Cat 2752, Lot 5) antibodies were purchased from Cell Signaling. Anti-GAPDH (clone mAbcam 9484, Cat ab9484, Lot 39238413-1) antibody were purchased from Abcam. Anti-phospho-Lck (Y394) monoclonal antibody (clone 7551-3, Cat MAB7500, Lot CHAZ0112071) was purchased from Bio-Techne. Homemade anti-DUSP22 antibody (DUSP22-C) was generated by immunization of mice with peptides (murine DUSP22 epitope: 181RPSSRRWSSFSTLPPLTYNNYTTET205). Homemade anti-DUSP22 monoclonal antibody (clone C8) was generated by immunization of mice with peptides (DUSP22 epitope: 170GKYKEQGRTEPQPGARRWSS189). Homemade anti-phospho-ERK1/2 (T202/Y204) antibody was generated by immunization of mice with peptides (murine p-ERK epitope: 133HTGFLTEpYVATRW145). Homemade anti-ERK1/2 (T202/Y204) antibody was generated by immunization of mice with peptides (murine ERK epitope: 308HPYLEQYYDPSDEPIAEAP326). Anti-CD3? (Y142) antibody (Cat A02421Y142, Lot A02421Y142) was purchased from Boster Biotechnology. Anti-phospho-LAT (Y191) (Cat 07-278, Lot 22138) and anti-LAT (clone 11B.12, Cat 05-770, Lot 26805) antibodies were purchased from EMD Millipore. Anti-PLCγ1 monoclonal antibody (clone B-6-4, Cat 05-366, Lot 23344) was purchased from Merck. For immunoblots, all primary antibodies were used at a 1:1,000 dilution, except for anti-Myc antibody (1:100,000). For PLA assays, all primary antibodies were used at a 1:300 dilution, except for anti-Flag and anti-Myc antibody (1:1,000). The antibodies for flow cytometry—including anti-mCD3?-FITC (clone 145-2C11, Cat 100306, Lot B151259), Anti-F4/80-PE/Cy7 (clone BM8, Cat 123114, Lot B207313) and anti-IgM-APC (clone RMM-1, Cat 406509, Lot B197034) antibodies —were purchased from BioLegend. Anti-mCD4-pacific blue (clone RM4-5, Cat 558107, Lot 2202191), anti-CD8-APC-Cy7 (clone 53-6.7, Cat 557654, Lot 1008430), anti-CD11b-FITC (clone M1/70, Cat 553310, Lot 74545) and anti-CD45R/B220-PE/Cy7 (clone RA3-6B2, Cat 552772, Lot B144210) antibodies were purchased from BD Biosciences. Anti-Foxp3-FITC monoclonal antibody (clone FJK-16s, Cat 53-5773-82, Lot 2416220) was purchased from Invitrogen. All antibodies for flow cytometry were used at 1:50 or 1:100 dilutions. The anfibodies for T cell activation, anti-CD3 (clone OKT3, Cat 317315, Lot B144612) antibody was purchased from BioLegend. Anti-CD3 (clone 145-2C11, Cat 553057, Lot 1116065), anti-CD3 (clone 500A2, Cat 553239, Lot 0314175), and anti-CD28 (clone 37.51, Cat 553294, Lot 1039452) antibodies were purchased from BD Pharmingen.

Validation

All primary antibodies used in this study are commercially available and validated by the manufacturers, except for anti-LAT (clone 11B.12, Cat 05-770), homemade anti-DUSP22 (DUSP22-C), anti-phospho-ERK1/2 (T202/Y204), and anti-ERK1/2 (T202/Y204) antibodies validated by Tan's group and reported in Li et al Nature Communications, 5, 1-13 (2014). Homemade anti-DUSP22 monoclonal antibody (clone C8) was validated and reported in Chen et al BMC Medicine 21, 46 (2023).

The validation information for the commercial antibodies are as follows. Flag (clone M2, Cat F3165), https://www.sigmaaldrich.com/TW/en/product/sigma/f3165?gclid=CjwKCAiApuCrBhAuEiwA8VJ6JqAzXT6Kly2SGh8rIvCGqtjFm2oqML48Ln7PT-sjlMHzRJodt9ExtxoCKSwQAvD_BwE. Myc (clone 9E10, Cat M4439), https://www.sigmaaldrich.com/TW/en/product/sigma/m4439. Lys48-specific ubiquitin (clone Apu2, Cat 05-1307) https://www.sigmaaldrich.com/TW/en/product/mm/051307. Lys63-specific ubiquitin (clone HWA4C4, Cat 05-1313), https://www.sigmaaldrich.com/TW/en/product/mm/051313. Vinculin (clone VIIF9, Cat MAB3574), https://www.merckmillipore.com/TW/zh/product/Anti-Vinculin-Antibody-clone-VIIF9-7F9,MM_NF-MAB3574?ReferrerURL=https%3A%2F%2Fwww.google.com%2F. His (clone 6AT18, Cat SAB1305538), https://www.sigmaaldrich.com/TW/en/product/sigma/sab1305538. CUL1 (clone D5, Cat sc-17775), https://www.scbt.com/p/cul-1-antibody-d-5. UBR2 (Cat PA5-37161), https://www.thermofisher.com/antibody/product/UBR2-Antibody-Polyclonal/PA5-37161. Tubulin (clone BT7R, Cat MA5-16308), https://www.thermofisher.com/antibody/product/beta-Tubulin-Loading-Control-Antibody-clone-BT7R-Monoclonal/MA5-16308). GST (clone DG122-2A7, Cat 05-311), https://www.sigmaaldrich.com/TW/en/product/mm/05311. HA (clone C29F4, Cat 3724S), https://www.cellsignal.com/products/primary-antibodies/ha-tag-c29f4-rabbit-mab/3724. Phospho-ZAP70 (Y319) (Cat 2701) https://

www.cellsignal.com/products/primary-antibodies/phospho-zap-70-tyr319-syk-tyr352-antibody/2701. ZAP70 (Y493) (Cat 2704) https://www.cellsignal.com/products/primary-antibodies/phospho-zap-70-tyr493-syk-tyr526-antibody/2704. ZAP70 (clone 99F2, Cat 2705) https://www.cellsignal.com/products/primary-antibodies/zap-70-99f2-rabbit-mab/2705. Phospho-LAT (Y171) (Cat 3581) https://www.cellsignal.com/products/primary-antibodies/phospho-lat-tyr171-antibody/3581. Phospho-PLCγ1 (Y783) (Cat 2821) https://www.cellsignal.com/products/primary-antibodies/phospho-plcg1-tyr783-antibody/2821. βTrCP (clone D13F10, Cat 4394) https://www.cellsignal.com/products/primary-antibodies/b-trcp-d13f10-rabbit-mab/4394. Lck (Cat 2752) (https://www.cellsignal.com/products/primary-antibodies/lck-antibody/2752). GAPDH (clone mAbcam 9484, Cat ab9484) https://www.abcam.com/products/primary-antibodies/gapdh-antibody-mabcam-9484-loading-control-ab9484.html). Phospho-Lck (Y394) (clone 7551-3, Cat MAB7500) https://www.rndsystems.com/products/human-phospho-lck-y394-antibody-755103_mab7500?gad_source=1&gclid=CjwKCAiApuCrBhAuEiwA8VJ6Jjif_0EvbNDChqFlbTNq-duUO7GsWpEQYatPyeXhO06RAXMmc-_qYBoCE44QAvD_BwE&gclsrc=aw.ds. CD3? (Y142) (Cat A02421Y142) https://www.bosterbio.com/anti-phospho-cd3-zeta-y142-cd247-antibody-a02421y142-boster.html. CD3e (clone 6B10.2, Cat sc-1239) https://www.scbt.com/p/cd3-zeta-antibody-6b10-2. Phospho-LAT (Y191) (Cat 07-278) https://www.merckmillipore.com/TW/zh/product/Anti-phospho-LAT-Tyr191-Antibody,MM_NF-07-278?ReferrerURL=https%3A%2F%2Fwww.google.com%2F. PLCγ1 (clone B-6-4), https://www.merckmillipore.com/TW/zh/product/Anti-PLC-1-Antibody-clone-B-6-4,MM_NF-05-366?ReferrerURL=https%3A%2F%2Fwww.google.com%2F.

mCD3-FITC (clone 145-2C11), F4/80-PE/Cy7 (clone BM8), anti-IgM-APC (clone RMM-1), mCD4-pacific blue (clone RM4-5), CD8-APC-Cy7 (clone 53-6.7), anti-CD11b-FITC (clone M1/70), CD45R/B220-PE/Cy7 (clone RA3-6B2), and Foxp3-FITC (clone FJK-16s): FC (flow cytometry) were listed under “Application” and noted with “Quality tested” (referring to that “each lot of this antibody is quality control tested”), and mouse is listed under “Verified Reactivity” (https://www.biolegend.com/en-gb/clone-search/fitc-anti-mouse-cd3epsilon-antibody-23?GroupID=BLG248; https://www.biolegend.com/de-at/clone-search/pe-cyanine7-anti-mouse-f4-80-antibody-4070?GroupID=GROUP20; https://www.biolegend.com/ja-jp/products/apc-anti-mouse-igm-2335?GroupID=GROUP23; https://www.bdbiosciences.com/en-eu/products/reagents/flow-cytometry-reagents/research-reagents/single-color-antibodies-ruo/pacific-blue-rat-anti-mouse-cd4.558107; https://www.bdbiosciences.com/en-us/products/reagents/flow-cytometry-reagents/research-reagents/single-color-antibodies-ruo/apc-cy-7-rat-anti-mouse-cd8a.561967; https://www.bdbiosciences.com/en-us/products/reagents/flow-cytometry-reagents/research-reagents/single-color-antibodies-ruo/fitc-rat-anti-cd11b.553310; https://www.bdbiosciences.com/en-us/products/reagents/flow-cytometry-reagents/research-reagents/single-color-antibodies-ruo/pe-cy-7-rat-anti-mouse-cd45r-b220.561881; https://www.thermofisher.com/antibody/product/FOXP3-Antibody-clone-FJK-16s-Monoclonal/11-5773-82).

anti-CD3 (clone OKT3, Cat 317315), anti-CD3 (clone 145-2C11, Cat 553057), anti-CD3 (clone 500A2, Cat 553239), and anti-CD28 (clone 37.51, Cat 553294) were listed under “Application” and noted with “Quality tested” or "quality certification" (referring to that “each lot of this antibody is quality control tested”), and “Verified Reactivity” were noted on the websites (https://www.biolegend.com/de-at/search-results/purified-anti-human-cd3-antibody-3642?GroupID=BLG4203&Clone=OKT3, https://www.bdbiosciences.com/en-au/products/reagents/flow-cytometry-reagents/research-reagents/single-color-antibodies-ruo/purified-na-le-hamster-anti-mouse-cd3e.553057, https://www.bdbiosciences.com/en-au/products/reagents/flow-cytometry-reagents/research-reagents/single-color-antibodies-ruo/purified-na-le-hamster-anti-mouse-cd3e.553057,https://www.bdbiosciences.com/en-au/products/reagents/flow-cytometry-reagents/research-reagents/single-color-antibodies-ruo/purified-hamster-anti-mouse-cd3e.553238, https://www.bdbiosciences.com/en-us/products/reagents/flow-cytometry-reagents/research-reagents/single-color-antibodies-ruo/purified-na-le-hamster-anti-mouse-cd28.553294).

Eukaryotic cell lines

Policy information about cell lines and Sex and Gender in Research

Cell line source(s)	HEK293T, Human Jurkat (E6-1 clone) (American Type Cell Culture, TIB-152) T leukemia, their derivative J.Cam1.6 clone (Lck deficient) (American Type Cell Culture, CRL-2063), and J-TAg clone cells were from the American Type Culture Collection (ATCC, USA) and cultured under standard conditions.
Authentication	The cell lines were authenticated by ATCC via STR profiling.
Mycoplasma contamination	Cells were regularly monitored for mycoplasma contamination. All cell lines tested negative for mycoplasma contamination.
Commonly misidentified lines (See ICLAC register)	No commonly misidentified cell lines were used.

Animals and other research organisms

Policy information about studies involving animals; ARRIVE guidelines recommended for reporting animal research, and Sex and Gender in Research

Laboratory animals	C57BL/6, JKAP knockout, UBR2 knockout mice were generated from Transgenic Mouse Core of NHRI. JKAP knockout mice were bred with UBR2 homozygous knockout mice or with UBR2 heterozygous knockout to generate JKAP/UBR2 double knockout mice or JKAP knockout/UBR2 heterozygous knockout mice. Mice of both male and female at different ages according to experimental design were housed in IACUC approved conditions. Sex-matched, 12- to 24-week-old C57BL/6J wild-type, UBR2 knockout, and DUSP22 knockout, DUSP22/UBR2 double knockout, DUSP22-/-;UBR2+/- mice were used in this study. Mice were bred and housed at the specific-pathogen-free cages with at most 4 animals per cage. These mice were maintained in a 12 h light/12 h dark cycle, and the housing temperature and humidity were maintained at 24 °C and 50%, respectively. All mice were euthanized by CO2 inhalation.
Wild animals	No wild animals were involved in this study.
Reporting on sex	Aged- and sex-matched mice were used in this study. Female mice were used for EAE induction.

Field-collected samples	No field collected samples were used in the study.
Ethics oversight	All mouse experiments were performed in the Association for Assessment and Accreditation of Laboratory Animal Care International (AAALAC)–accredited animal housing facilities at the National Health Research Institutes (NHRI). All mice were used according to the protocols and guidelines approved by the Institutional Animal Care and Use Committee of NHRI.

Note that full information on the approval of the study protocol must also be provided in the manuscript.

Clinical data

Policy information about clinical studies

All manuscripts should comply with the ICMJE guidelines for publication of clinical research and a completed CONSORT checklist must be included with all submissions.

Clinical trial registration	Not applicable as this was not a clinical trial.
Study protocol	The study protocol was described in the manuscript.
Data collection	All healthy control and SLE patients were recruited from May 2022 to February 2023. Peripheral blood from healthy controls and SLE patients were collected up to February 2023.
Outcomes	Because this was an observational study, there were no primary or secondary outcome.

Plants

Seed stocks	n/a
Novel plant genotypes	n/a
Authentication	n/a

Flow Cytometry

Plots

Confirm that:

- ☒ The axis labels state the marker and fluorochrome used (e.g. CD4-FITC).
- ☒ The axis scales are clearly visible. Include numbers along axes only for bottom left plot of group (a 'group' is an analysis of identical markers).
- ☒ All plots are contour plots with outliers or pseudocolor plots.
- ☒ A numerical value for number of cells or percentage (with statistics) is provided.

Methodology

Sample preparation	Thymocytes, splenocytes, lymph node cells, and bone-marrow-derived cells were harvested from wild-type and UBR2 knockout mice.
Instrument	FACS CantoII (BD Biosciences)
Software	FlowJo analytical software
Cell population abundance	No cell sorting procedure was used in this study.
Gating strategy	Cell debris were excluded by SSC and FSC gating. Gating strategy was shown in Supplementary Fig. 5.

- ☒ Tick this box to confirm that a figure exemplifying the gating strategy is provided in the Supplementary Information.
